# Identification of novel recombinants and proposed standard reference genomes for phylogenetic classification of canine parvovirus-2 (CPV-2): Comprehensive analysis revealing global evolutionary trait

**DOI:** 10.3389/fvets.2022.1030522

**Published:** 2022-11-15

**Authors:** Amina Nawal Bahoussi, Pei-Hua Wang, Zi-Hui Ma, Nikita Rani, Changxin Wu, Li Xing

**Affiliations:** ^1^Institutes of Biomedical Sciences, Shanxi University, Taiyuan, China; ^2^The Key Laboratory of Medical Molecular Cell Biology of Shanxi, Shanxi University, Taiyuan, China; ^3^Shanxi Provincial Key Laboratory for Prevention and Treatment of Major Infectious Diseases, Taiyuan, China; ^4^The Key Laboratory of Chemical Biology and Molecular Engineering of Ministry of Education, Shanxi University, Taiyuan, China

**Keywords:** canine parvovirus 2 (CPV-2), genotyping, genetic evolution, recombination, parsimony-informative sites

## Abstract

Sustained spread and continuous evolution of CPV-2 generate new genetic information; nevertheless, there is no adopted phylogenetic tool, and parvo virologists still refer to the three antigenic variants. Herein, this report attempted to review the evolutionary trait of CPV-2 and proposed standard reference genomes using the Maximum Likelihood-based phylogenetic analysis and Parsimony-Informative Sites. The analysis revealed three main evolutionary pathways where CPV-2 strains cluster into distinct clades depicted as GI, GII, or GIII, respectively. Furthermore, novel CPV-2 natural recombinants were detected, occurring only between the newly identified strains (2017–2020). Those findings provide unique insights into the evolutionary relatedness of CPV-2, clarify discrepancies between different geographic areas and will contribute to achieving a more reliable CPV-2 genetic and evolutionary genotyping classification.

## Introduction

Parvoviruses are small ubiquitous DNA viruses that infect multiple varieties of animal species, including humans. *Carnivore protoparvovirus 1* is a specie within the genus Protoparvovirus of the *Parvoviridae* family and one of the eight genera that make up the Parvovirinae subfamily (1). Canine parvovirus is a small non-enveloped, linear negative-sense single-stranded DNA virus that affects the dog population and other mammals such as wolves and raccoons. The viral genome is about 5.2 kb in length and consists of two major open reading frames (ORF1 and ORF2) (2). ORF1 encodes the nonstructural proteins: NS1 and NS2, responsible for the viral replication and the DNA packaging (2), while ORF2 encodes the capsid proteins: VP1 and VP2, which play a prominent role in the virus-host interaction and represent the main antibody inducers (2, 3). The capsid protein genes harbor the most substitutions that distinguish the canine parvoviruses from the feline panleukopenia virus (4). The VP1 coding sequence encompasses the complete VP2 genomic coding region. CPV-2 infection is characterized by two main clinical forms, including gastroenteritis with diarrhea and vomiting in dogs of all ages (5) and heart failure with myocarditis in pups below 3 months (6, 7).

CPV outbreaks in dogs emerged in the mid of 1970s. The virus was recognized for the first time in 1978 and was designated CPV-2 (8). In the 1980s, the original CPV-2 acquired a number of mutations, leading to the emergence of a novel CPV-2a with three amino acid substitutions in VP2 protein (M87L, A300G, and D305Y) (9), followed by CPV-2b, which differs from the CPV-2a at the VP2 residues N426D and I555V (10). Another antigenic variant type, CPV-2c (D426E), appeared first in Germany in 1996 (11) and was isolated from domestic dogs in Italy in 2000 (12) and then in Vietnam (13). The prophylactic vaccination is the main preventive measure for CPV-2 infection in domestic dog populations. CPV-2 vaccination is safe and is based on CPV-2 or CPV-2b (14). However, despite the availability of vaccines, a sustained spread of CPV-2 infection and outbreaks among fully vaccinated dogs have been reported (15). The emergence of new CPV-2 variants raised concerns and questioned to what extent the cross-protection by licensed MLV type CPV-2 or CPV-2b can elicit protection against CPV-2c (15, 16).

Currently, the geographical distribution of CPV-2 (2a, 2b, 2c) demonstrates discrepancies among different areas, with limited spatial spread among populations (17, 18). The phylogenetic analysis revealed that both CPV-2a and CPV-2b viruses are polyphyletic, suggesting a more complex antigenic evolution (18). Recently, the antigenic classification and the partial sequencing of VP2 have been reported as insufficient to clarify the epidemiological scenario of CPV-2, and multiple studies have attempted to provide explicit phylogenetic frameworks for the relationship between CPV-2 variants and their genetic evolution, which remains challenging (17, 19, 20). Therefore, the present study aims to propose standardized CPV-2 reference genomes to help characterize the genetic evolution of CPV-2 strains and develop a unified genotyping molecular classification tool.

## Materials and methods

### Dataset

The complete coding nucleotide sequences of 319 CPV-2 in the final dataset (nt 4269 in length) retrieved from the NCBI GenBank database (https://www.ncbi.nlm.nih.gov/) were detected from dogs and other animals such as felines, racoons, Paradoxurus musangus, yak, pangolin, American mink, river otter, and common raccoon dogs between 1978 and 2020 from all continents, including twenty-six worldwide countries: North America (the USA, Canada), South America (Uruguay, Brazil, Argentina, Paraguay, Ecuador), Europe (Germany, Italy, France, Albania, Finland, Russia), Africa (Nigeria), Asia (China, Japan, India, Vietnam, Singapore, Mongolia, Thailand, South Korea, Iran, and Bangladesh), and Oceania (New Zealand, Australia). The sequences were aligned with Clustal Omega (https://www.ebi.ac.uk/Tools/msa/clustalo/) (21), then analyzed. The viruses in this report are designated by their GenBank ID, name, country, year of collection and antigenic type in a format as [GenBank ID_Virus name (country-collection year-antigenic type in GenBank)].

### Phylogenetic analysis

The evolutionary history of CPV-2 was inferred using the Maximum Likelihood method and Tamura Nei model (22). The tree with the highest log likelihood (−17,318.14) is shown. The percentage of trees in which the associated taxa clustered together is shown next to the branches. Initial trees for the heuristic search were obtained automatically by applying Neighbor-Join and BioNJ algorithms to a matrix of pairwise distances estimated using the Tamura-Nei model. Topologies with superior log-likelihood values are selected. The numbers at each branch indicate the percentage of bootstrap values of 1,000 replicates. Trees are drawn to scale, with branch lengths measured in the number of substitutions per site. The evolutionary analyses involving CPV-2 complete nucleotide sequences were conducted in Molecular Evolutionary Genetics Analysis software version 11 (MEGA 11) (23).

### Parsimony-informative site analysis (PIS)

Parsimony-informative sites (PIS) were analyzed to identify and compare the mutation at the protein sequence level (NS1, NS2, and VP1/VP2 proteins) from four different geographic areas (Italy, China, the USA, and Uruguay). A site is parsimony informative (PI) if it contains at least two types of amino acids that display at least two distinct character states, each of which occurs in at least two of these sequences as a minimum frequency.

### Recombination detection

To assess the occurrence of recombination events, the 319 CPV-2 full-length genomes included in our dataset were analyzed using the RDP4 software package (24). The recombinants were detected by each of the seven algorithms, including RDP, GENECONV, Bootscan, MaxChi, Chimera, SiScan, and 3seq embedded in the RDP4 package. Furthermore, phylogenetic trees of the genomes involved in the identified recombination events were built using the neighbor-joining method based on the indicated nucleotide genomic regions of CPV-2 strains in the MEGA 11 (23). Several viral genomes not involved in the recombination were included as references. The recombination breakpoint distribution map was generated based on the nucleotide sequences (~4269 nt) of each detected recombinant using the RDP4 software. The genomic similarity between the identified CPV-2 strains involved in recombination (parental and recombinant sequences) and Bootscanning analyses were carried out using Simplot ver. 3.5.1 (25).

## Results

### Proposed reference genomes for CPV-2 phylogenetic analysis

**Figure 1:**
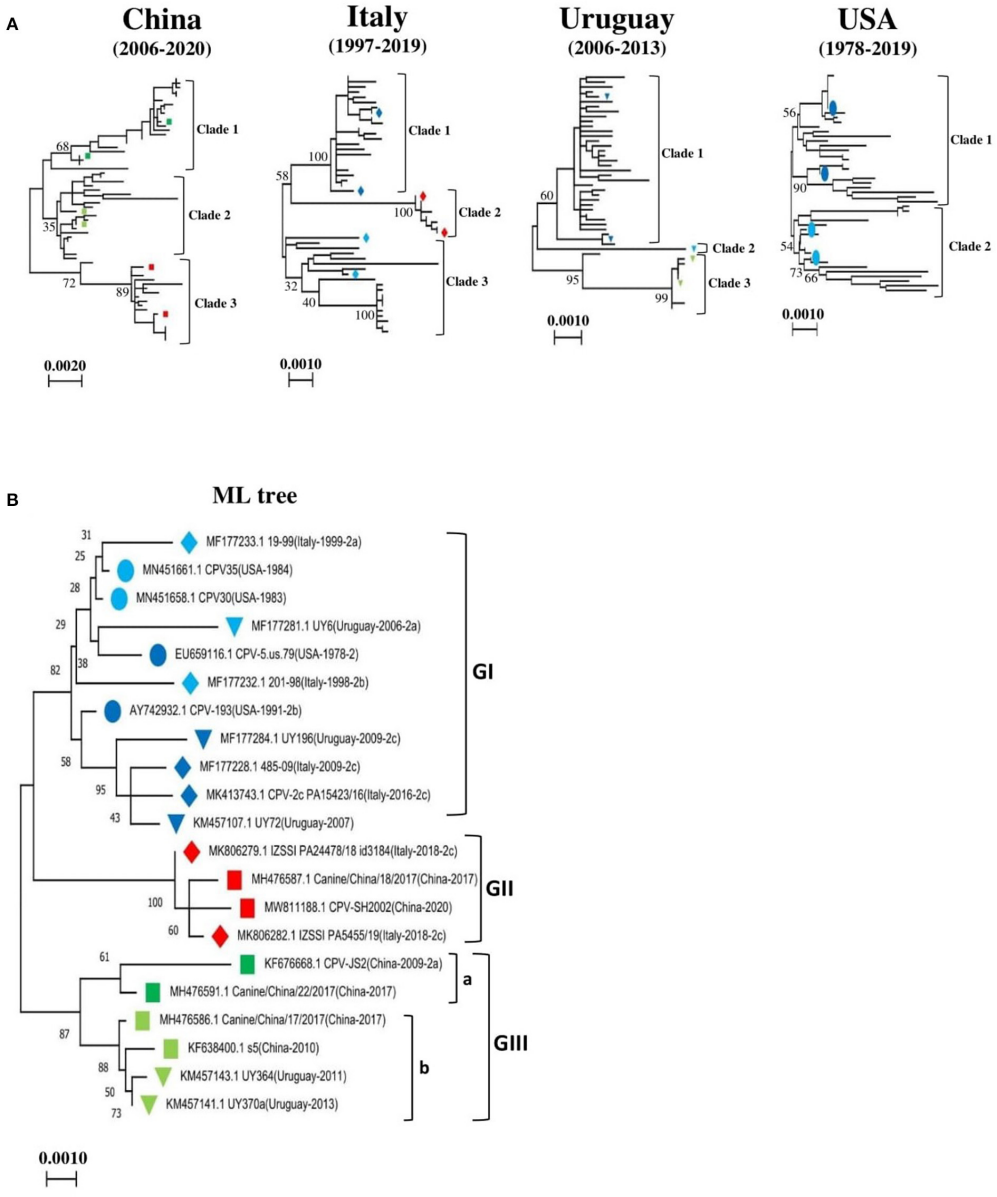
Country-based phylogenetic trees of the entire genome sequences of CPV-2 strains. **(A)** Maximum-Likelihood (ML)-based trees of CPV-2 strains from four main countries: China (*n* = 62), Italy (*n* = 50), the USA (*n* = 47), and Uruguay (*n* = 48). Trees were generated using MEGA 11 software. Three main clades in phylogenetic trees of China, Italy and Uruguay are shown (clade1, 2 and 3), and two main clades (clades 1 and 2) in the USA. **(B)** Consensus phylogenetic tree of twenty-one standard reference sequences of CPV-2 strains. The standard references correspond to representative CPV-2 strains from each phylogenetic tree clade of **(A)**. The numbers at each branch indicate the percentage of bootstrap values of 1,000 replicates. The scale bars reveal the number of inferred substitutions per site. The CPV-2 strains are identified in a format as [GenBank ID_virus name (country-collection year-antigenic type in GenBank)]. Viruses from China, Italy, Uruguay, and the USA are indicated with Square, Diamond, Triangle, and Circle, respectively. Blue, red, and green colors indicate strains belonging to GI, GII, and GIII, respectively.

The complete nucleotide sequences of 319 CPV-2 detected from dogs and other animals between 1978 and 2020 from all continents, including North and South America, Africa, Europe, Asia, and Oceania, were included in this analysis. The phylogenetic relatedness for the entire CPV-2 genomes was analyzed using the Maximum Likelihood (ML) approach (Figure 1A, Supplementary Figures 1A–D) in MEGA 11 software. First, we segregated our data into four main groups, in which CPV-2 full-length sequences of the four major countries that dominated our dataset, including China (62), Italy (50), Uruguay (48), and the USA (47), were grouped and analyzed using the ML approach (Figure 1A). The tree topology of CPV-2 full-length sequences detected in China between (2006–2020), in Italy between (1997–2019), and in Uruguay between (2006–2013) revealed that CPV-2 strains cluster into three main clades (clade1, 2, and 3) (Figure 1A, Supplementary Figures 1A–C); meanwhile the analysis of CPV-2 strains detected between (1978–2019) in the USA grouped only into two main clades (clade1 and clade2) (Figure 1A, Supplementary Figure 1D). Subsequently, we constructed a reliable ML-based phylogenetic tree comparing the evolution of CPV-2 full-length sequences from the different countries mentioned above together. To this intent, we selected two country-representative strains from each clade (clade1, 2, 3) of the individual phylogenetic trees of the four main countries′ data (with the exception that only one strain from clade 2 of viruses identified in Uruguay) (Figure 1A). In total, we selected twenty-one full-length references (Table 1) and found that CPV-2 strains cluster into three main divergent phylogenetic clades depicted as Genotype I, Genotype II and Genotype III (GI and GII, and GIII) (Figure 1B). Genotype III is further divided into two sub-genotypes (GIII-a and GIII-b) (Figure 1B). The phylogenetic tree comparing CPV-2 sequences indicates that CPV-2 can be divided into 3 different groups with different evolutionary biology in specific countries (USA, Uruguay, China, and Italy). Interestingly, the comparative ML-based tree (Figure 1B) revealed that all CPV-2 representative strains detected in the USA cluster into the GI clade, while other strains from Italy: 485-09 (GenBank ID: MF177228), PA15423/16 (GenBank ID: MK413743) from clade1, and 201-98 (GenBank ID: MF177232), 19-99 (GenBank ID: MF177233) from clade 3 (Figure 1A, Supplementary Figure 1B), and from Uruguay: UY196 (GenBank ID: MF177284) and UY72 (GenBank ID: KM457107) from clade 1, and UY6 (GenBank ID: MF177281) from clade 2 (Figure 1A, Supplementary Figure 1C), cluster into GI (Figure 1B). As shown in Figure 1B, the GI clade encompasses CPV-2 strains regardless of their antigenic classification. Furthermore, CPV-2 sequences detected in China, including CPV-SH2002 (GenBank ID: MW811188) and Canine/China/18/2017 (GenBank ID: MH476587) from clade 3 (Figure 1A, Supplementary Figure 1A), cluster into GII (Figure 1B) along with Italy CPV-2 strains IZSSI PA24478/18 id 3184 (GenBank ID: MK806279) and IZSSI PA5455/19 (GenBank ID: MK806282) from clade 2 (Figure 1A, Supplementary Figure 1B). The cluster GII (Figure 1B) indicates a distinctive evolutionary feature of China CPV-2 strains, which could be unequivocally linked to Italy CPV-2 (clustering under the same GII clade) (Figure 1B). CPV-2 strains from China, belonging to clade 1: CPV-JS2 (GenBank ID: KF676668), Canine China/22/2017 (GenBank ID: MH476591) and clade 2: s5 (GenBank ID: KF638400), Canine/China/17/ 2017 (GenBank ID: MH476586) (Figure 1A, Supplementary Figure 1A), cluster into GIII under GIII-a and GIII-b sub-genotypes, respectively (Figure 1B). Meanwhile, CPV-2 strains from Uruguay, clade 2: UY364 (GenBank ID: KM457143), UY370 (GenBank ID: KM457141) (Figure 1A, Supplementary Figure 1C) cluster into GIII under GIII-b sub-genotype (Figure 1B). Therefore, our phylogenetic tree demonstrates the existence of three main pathways in the worldwide evolution of representative CPV-2 strains depicted as GI, GII, and GIII, in which China strains and the USA strains showed distinct routes, and GII encompasses mainly the newly CPV-2 strains identified between (2014–2020).

**Table 1:** Reference sequences of CPV-2 genotypes and subtypes.

**Genotype**	**Subtype**	**Antigenic subtype**	**GenBank ID**	**Strain**	**Country**	**Continent**	**Year of collection**
GI		2a	MN451658.1	CPV30	USA	America	1983
		2a	EU659116.1	CPV-5.us.79	USA	America	1978
		2b	AY742932.1	CPV-193	USA	America	1991
		2a	MN451661.1	CPV35	USA	America	1984
		2c	MF177284.1	UY196	Uruguay	America	2009
		2a	MF177281.1	UY6	Uruguay	America	2006
		2c	KM457107.1	UY 72	Uruguay	America	2007
		2a	MF177233.1	19-99	Italy	Europe	1999
		2b	MF177232.1	201-98	Italy	Europe	1998
		2c	MF177228.1	485-09	Italy	Europe	2009
		2c	MK413743.1	CPV-2Cpa15423/16	Italy	Europe	2016
GII		2c	MK806279.1	IZSSI PA24478/18 id3184	Italy	Europe	2018
		2c	MH476587.1	Canine/China/18/2017	China	Asia	2017
		2c	MW811188.1	CPV-SH2002	China	Asia	2020
		2c	MK806282.1	IZSSI PA5455/19	Italy	Europe	2018
GIII	GIII-a	2a	KF676668.1	CPV-JS2	China	Asia	2009
		2a	MH476591.1	Canine/China/22/2017	China	Asia	2017
	GIII-b	2a	MH476586.1	Canine/China/17/2017	China	Asia	2017
		2a	KF638400.1	s5	China	Asia	2010
		2a	KM457143.1	UY364	Uruguay	America	2011
		2a	KM457141.1	UY370a	Uruguay	America	2013
	GIII-c	2a	MN451689.1	CPV616	Nigeria	Africa	2018
	GIII-d	2a	MK144546.1	K01708-3	South Korea	Asia	2017

### Genotyping CPV-2 identified worldwide

**Figure 2:**
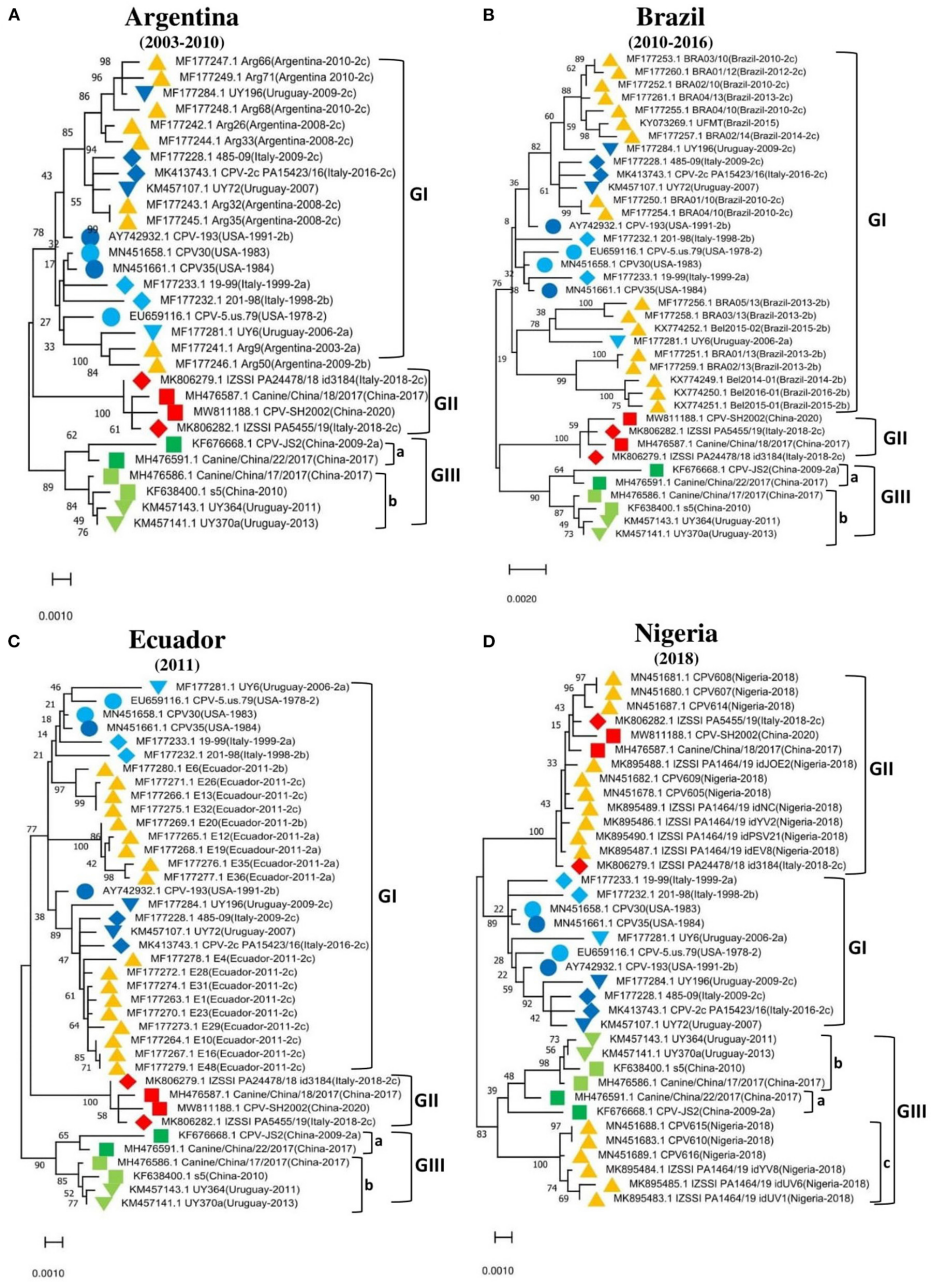
Phylogenetic analysis based on the full-length genome of CPV-2 strains from different regions. **(A)** Argentina (*n* = 9), **(B)** Brazil (*n* = 17), **(C)** Ecuador (*n* = 18), **(D)** Nigeria (*n* = 10). Trees were generated using the Maximum-Likelihood approach and MEGA 11 software. The numbers at each branch indicate the percentage of bootstrap values of 1,000 replicates. The evolutionary distances were computed using the Maximum Composite Likelihood method. The scale bar reveals the number of inferred substitutions per site. The CPV-2 strains are identified in a format as [GenBank ID_Virus name (country-collection year-antigenic type in GenBank)]. Yellow triangles indicate the strains from the country concerned by the analysis. Blue, red, and green colors indicate the standard reference sequences.

**Figure 3:**
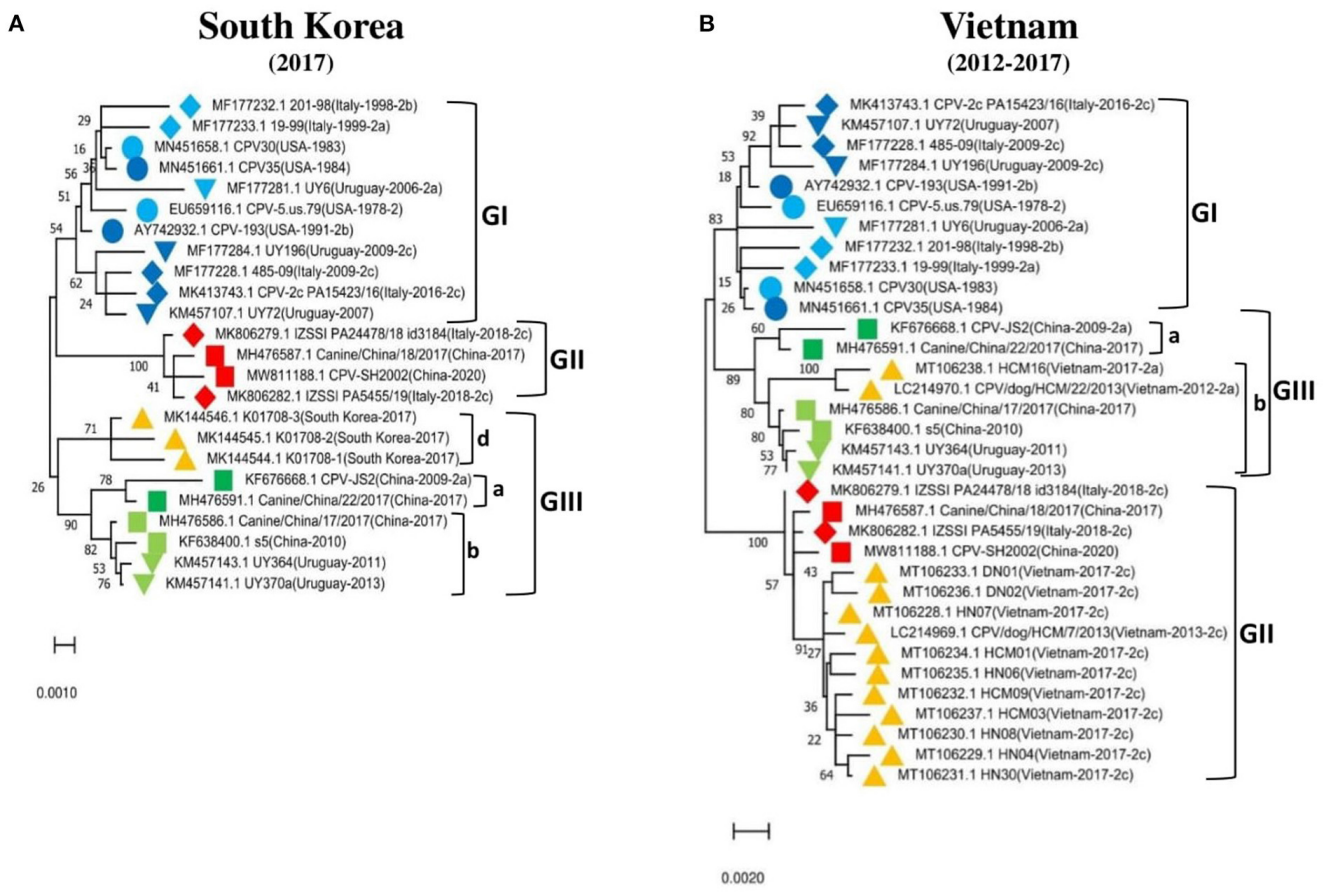
Phylogenetic analysis based on the full-length genome of CPV-2 strains from **(A)** South Korea (*n* = 3), **(B)** Vietnam (*n* = 13). Trees were generated using the Maximum-Likelihood approach and MEGA 11 software. The numbers at each branch indicate the percentage of bootstrap values of 1,000 replicates. The evolutionary distances were computed using the Maximum Composite Likelihood method. The scale bar reveals the number of inferred substitutions per site. The CPV-2 strains are identified in a format as [GenBank ID_Virus name (country-collection year-antigenic type in GenBank)]. Yellow triangles indicate the strains from the country concerned by the analysis. Blue, red, and green colors indicate the standard reference sequences.

**Figure 4:**
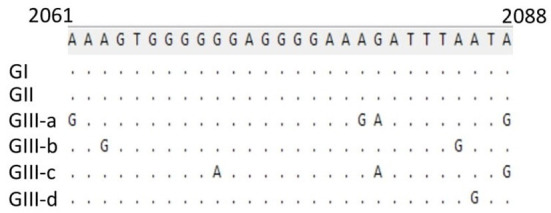
Genetic characteristics of viruses belonging to GIII (a, b, c, and d). The partial intron of the VP1 gene (nt 2061-2088) is conserved for GI and GII but indicated to be highly diversly variable for GIII-a, b, c, and d. Numbers indicate the nucleotide position relative to the genome of virus strain CPV6, GenBank ID: MN451653.1.

To further support these findings, we used the proposed standard references to classify strains detected in different countries worldwide, including Argentina, Brazil, Ecuador, South Korea, and Vietnam. Comparison of CPV-2 viral genomes detected in South America (e.g., nine from Argentina between 2003–2010, seventeen from Brazil between 2010–2016, and eighteen from Ecuador in 2011) independently revealed that strains from each of those South American countries cluster into GI (Figures 2A–C) along with the viruses detected in the USA and the most of Europe countries (Table 2, Supplementary Table 1). Comparison of Asia strains exhibited thirteen strains from Vietnam cluster into GII and GIII-b, while three strains from South Korea cluster into GIII-d (Figures 3A,B) along with the viruses detected in China and most of Asian countries (Table 2, Supplementary Table 1). CPV-2 sequences detected from Nigeria in Africa in 2018 (ten strains) cluster into GII and GIII-c (Figure 2D). Furthermore, a comparison of all the available CPV-2 full-length genomes reveals the genetic characteristics distinguishing three CPV-2 genotypes (GI, GII, and GIII) (Table 3). The partial intron of VP1 gene (nt 2061-2088) is found to be highly diversly variable for the sub-genotypes of GIII (a, b, c, and d), but highly conserved for GI and GII (Figure 4). GI and GIII comprise all three CPV-2 antigenic variants (2a, 2b, and 2c), while GII comprises mainly the 2c variants with one 2a variant CPV/CN/LN1/2014 identified in China in 2014 (GenBank ID: KR002800.1).

**Table 2:** Geographic distribution of CPV-2 genotypes.

**CPV-2 genotype**		**Africa**	**Middle east**	**Europe**	**Asia**	**America**	**Oceania**
GI			Iran	Italy, France, Albania, Germany, Finland, Russia	Japan, India	Uruguay, USA, Paraguay, Argentina, Brazil, Canada, Ecuador	Australia, New Zealand
GII		Nigeria		Italy	China, Vietnam, Mongolia, Thailand		
GIII	a		Iran		China, Bangladesh, Singapore, India		
	b		Iran		China, Vietnam, India	Uruguay, Canada	
	c	Nigeria					
	d				South Korea		

**Table 3:** Genetic signature of CPV-2 genotypes.

**Genotype**	**nt 178 (aa 60 in NS1)**	**nt 1662 (aa 554 in NS1)**	**nt 1889 (aa 630 in NS1)**	**nt 2113 (intron of VP1 gene)**	**nt 3484-3485 (aa 324 in VP2)**
	Missense	Silent	Missense		Missense
GI	A (I)	T (H)	T (L)	A	TA (Y)
GII	G (V)	C (H)	C (P)	G	AT (I)
GIII	A (I)	T (H)	T (L)	A	AT (I)

nt, nucleotide position (relative to the genome of virus strain CPV6, GenBank ID: MN451653.1). aa, amino acid position relative to the NS1 or VP2 as indicated).

### The effect of parsimony-informative site (PI) on CPV-2 tree topology

**Figure 5:**
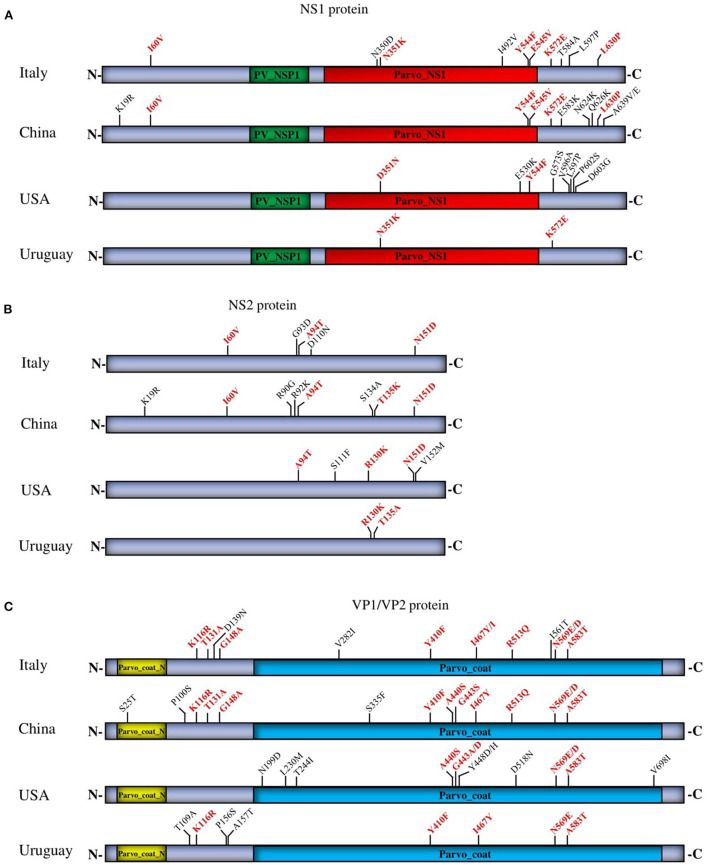
Parsimony analysis of NS1 **(A)**, NS2 **(B)**, and VP1/VP2 **(C)** proteins of circulating CPV-2 strains from four countries (China, Italy, the USA, and Uruguay). The common and unique PI sites are indicated in red and black letters and numbers, respectively.

Despite the stability and the lower rate of mutations in DNA viruses, the CPV-2 genome has shown significant nucleotide substitutions similar to that of RNA viruses, particularly in VP1/VP2 structural proteins used as an antigenic determinant (26). To compare the naturally occurring changes in the structural and nonstructural proteins, we proceeded with analyzing the parsimony-informative sites (PI), which are important in constructing phylogenetic trees, and found obvious differences between the four countries (China, Italy, the USA, and Uruguay). The extended PI analysis of multiple viral proteins (NS1, NS2, and VP1/VP2) in our report identified distinct molecular signatures of strains from each geographic area and helped to properly understand the spread of CPV-2 at a local level and in all continents. The PI substitutions involved are specific to China viruses (NS1: K19R, E583K, N624K, Q626K, A639V/E—NS2: K19R, R90G, R92K, S134A—VP1/VP2: S25T, P100S, S335F), to Italy viruses (NS1: N350D, I492V, T584A, L597P—NS2: G93D, D110N—VP1/VP2: D139N, V282I, I561T), USA viruses (NS1: E530K, G573S, V596A, L597P, P602S, D603G—NS2: S111F, V152M—VP1/VP2: N199D, L230M, T244I, Y448D/H, D5118N, V698I), and Uruguay viruses (NS1:—NS2:—VP1/VP2: T109A, P156S, A157T) (Figure 5). The analysis identified (I60V, N351K, Y544F, E545V, L630P, K572E, and D351N in NS1) (Figure 5A), (I60V, A94T, T135K, N151D, and R130K in NS2) (Figure 5B), and (K116R, T131A, G148A, Y410F, A440S, G443S, I467Y/I, R513Q, N569E/D, and A583T in VP1/VP2) (Figure 5C) as the common PI sites shared by viruses detected in at least two countries. PI site 569N/E/D in VP1/VP2 corresponds to 426 in VP2, which is used initially to categorize the antigenic type variant of CPV-2 (2a, 2b, and 2c) (Figure 5C). In NS1, most detected amino acid substitutions are shown at the C terminal region, whereas NS2 and VP1/VP2 substitutions are localized all over the coding regions. Even though some of the protein sequences exhibited similar PI sites (Figure 5), each country′s strains revealed distinct and unique PI sites (Figure 5) (China, Italy, the USA, and Uruguay), which are consistent with the results of the phylogenetic tree topology.

### Screening of CPV-2 potential recombinants

**Figure 6:**
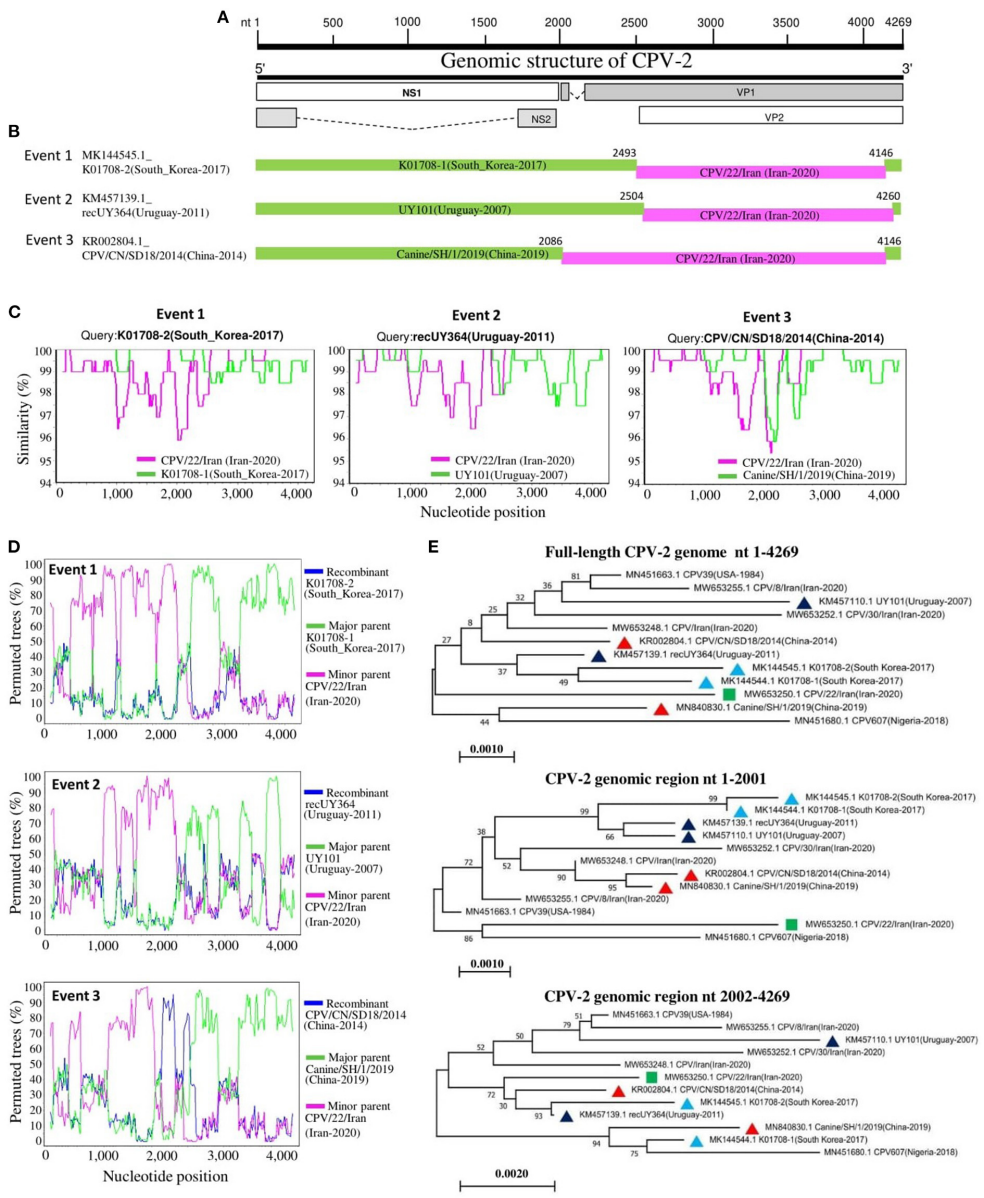
Recombination analysis of circulating CPV-2 genomes. **(A)** A schematic of the genome organization of CPV-2 is shown at the top of the figure. **(B)** Diagram of the genomes of recombinant viruses. The major parents are in green, while the minor parents are in pink. The numbers indicate the nucleotide positions (nt) of beginning and ending breakpoints of recombination and are relative to the genomic sequence of corresponding recombinant viruses. **(C)** Genomic similarity Simplot analysis of viruses involved in the three recombination events. **(D)** Bootscanning analysis of viruses involved in the three recombination events with the genome of CPV607 (GenBank ID: MN451680.1) as a non-recombinant reference. **(E)** Phylogenetic analysis of seven viruses involved in recombination events and five non-recombinant viral strains based on the full-length genome or the indicated genomic regions. The virus is indicated in the format of GenBank ID_virus name (collection country-collection date). The minor parent is indicated with a green square. The viruses detected in South Korea, Uruguay, and China are labeled with blue, black, or red triangles, respectively. The numbers at each branch indicate the bootstrap values (%) of 1,000 replicates. The scale bars indicate the number of inferred substitutions per site.

Recombination analysis of 319 full-length CPV-2 genomes was performed using the RDP4 software package (24). The recombination events were identified by each of the seven algorithms embedded in the RDP4 package, including RDP, GENECONV, Bootscan, MaxChi, Chimera, SiScan, and 3seq. The analysis describes the occurrence of intra- and intercontinental recombination among relatively stable CPV-2 DNA genomes, observed only in the newly identified CPV-2 circulating strains (Table 4). The virus CPV/22/Iran detected in Iran in 2020 (GenBank ID: MW653250.1, antigenic type CPV-2a) seems to be involved in all detected recombination events as a potential minor parent. The virus strains K01708-1 (GenBank ID: MK144544.1, antigenic type−2c) detected in South Korea, UY101 (GenBank ID: KM457110.1, antigenic type−2c) detected in Uruguay, and Canine/SH/1/2019 (GenBank ID: MN840830.1, antigenic type−2c) detected in China served as potential major parents involved in Event 1, Event 2, and Event 3, respectively (Table 4). The three recombinant strains K01708-2, recUY364, and CPV/CN/SD18/2014 belonged to CPV-2a (Table 4). The obtained results in recombination events might be linked to the economic relationship between Iran and other countries. We mapped the beginning and ending breakpoints of the three detected recombinant strains and located them at a similar coding region of the genome within the VP1/VP2 coding region (Figures 6A,B). To further confirm the recombination events, we next sought out additional evidence for the identified recombinants using Bootscanning and similarity analyses. The three recombination events were strongly supported by significant results of genomic similarity analysis using Simplot ver. 3.5.1 (25) and Bootscanning analysis. As shown in Figure 6C, the entire genome of recombinant K01708-2 exhibited a high genetic similarity with that of K01708-1, except the nucleotides 2500-4146, which showed a high genetic similarity with that of CPV/22/Iran (Figure 4C, Event 1). In Bootscanning analyses (Figure 6D), the genome of recombinant K01708-2 demonstrated the same percentage of permuted trees as the major parent K01708-1, except for the segment nt 2500-4146 of the genomes, which exhibited the same percentage of permuted trees with the minor parent CPV/22/Iran (Figure 6D, Event 1). Similar phenomena were also observed for recombination Event 2 and Event 3. Furthermore, the recombination network was verified by producing phylogenetic trees based on the CPV-2 full-length or individual fragments of the genome (Figure 6E). The fragment nt 1-2001 encodes NS1 and NS2. The fragment nt 2002-4269 encodes VP1and VP2, and the fragment nt 1-4269 represents the full-length CPV-2 genome. In the full-length genome-based phylogenetic tree, the viruses involved in recombination events were labeled with different symbols and sorted into different evolutionary branches. In the fragment nt 1-2001-based phylogenetic tree, the viruses detected in the same country (South Korea, Uruguay, or China) and involved in the same recombination event are genetically closer. However, in the fragment 2002-4269-based phylogenetic tree, three recombinants (K01708-2 in Event 1, recUY364 in Event 2, and CPV/CN/SD18/2014 in Event 3) fall in the same group as the common minor parent CPV/22/Iran.

**Table 4:** Identification of potential recombination events in the genome of CPV-2 using the RDP4 software package.

**Event Serial Number**	**Recombinant**		**Major Parent**		**Minor Parent**		**Detection Methods**		
	**GenBank ID_Virus Name (Country-Year)**	**Type, Host**	**GenBank ID_Virus Name (Country-Year)**	**Type, Host**	**GenBank ID_Virus Name (Country-Year)**	**Type, Host**	**R**	**G**	**B**	**M**	**C**	**S**	**T**
1	MK144545.1_K01708-2 (South_Korea-2017)	2a, dog	MK144544.1_K01708-1 (South_Korea-2017)	2c, dog	MW653250.1_CPV/22/Iran (Iran-2020) ^*^	2a, dog	+	–	+	+	–	+	+
2	KM457139.1_recUY364 (Uruguay-2011)	2a, dog	KM457110.1_UY101 (Uruguay-2007)	2c, dog	MW653250.1_CPV/22/Iran (Iran-2020) ^*^	2a, dog	–	+	+	+	+	+	+
3	KR002804.1_CPV/CN/ SD18/2014 (China-2014)	2a, dog	MN840830.1_Canine/SH/1/ 2019 (China-2019) ^*^	2c, dog	MW653250.1_CPV/22/Iran (Iran-2020) ^*^	2a, dog	-	-	+	+	-	+	+

The potential recombination events were identified by each of the seven algorithms (RDP, GENECONV, Bootscan, MaxChi, Chimera, SiScan, and 3seq) embedded in the RDP4 package.

R, RDP; G, GENECONV; B, BootScan; M, MaxChi; C, Chimera; S, SiScan; T, 3seq. +, verified; -, not verified.

* The major or minor parent may be the actual recombinant due to the possibility of misidentification.

## Discussion

Following the emergence of CPV-2 in 1977, several variants (2a, 2b, 2c) have been categorized based on the amino acid substitutions in the VP2 structural protein, which remains the only adopted antigenic classification tool till to date. Recently, researchers attempted to phylogenetically classify CPV-2 based on the entire genome or VP2 complete coding sequences (17, 19, 20). Grecco et al. suggested classifying CPV-2 based on the geographical distribution using a phylodynamic analysis of 144 CPV-2 genomes (CPV-2, CPV-2a), sixty-three of which were from South America and Europe and were belonging to the CPV-2a lineage (17). Grecco et al. classified CPV-2 strains into Europe clades (Eur-I and Eur-II), South America clade (SA-I), and Asia clade (Asia-I) (17). In contrast, Chung et al. (19) and de Oliveira Santana et al. (20) made the Bayesian phylogenetic analysis with significant inconsistency. Chung et al. analyzed 200 complete genome sequences, 200 VP1, 275 VP2, and 200 NS1 protein-coding genes, and classified CPV-2 into three major clades (I, II, and III) independently of the pathogen′s geographic origins (19). de Oliveira Santana et al. explored the distribution of CPV-2 in Brazil and the global phylogeny based on 298 complete genome sequences and 684 VP2 protein sequences and have divided the complete genomes into two main clades (I and II) (*n* = 284 CPV-2) while the VP2 sequences into four distinct clades named W1, W2, W3, and W4 (*n* = 644 VP2) (20). de Oliveira Santana et al. report has eliminated the geographic evolutionary scenario and indicated that each clade had samples from different continents with a distribution of the three antigenic variants (2a, 2b, 2c) (20). Other researchers have suggested “CPV-2a clade” as a single common clade of the three antigenic variants “CPV-2a, 2b, 2c”, that forms a distinct branch from that of the old strain CPV-2 (27). Our report builds upon theirs with twenty-one more full-length sequences in addition to the analyzed strains in de Oliveira Santana et al. report (20), using the ML method and parsimony-informative analysis (PI) to map the genomic changes and review the evolutionary traits of CPV-2. Recently, several studies debated the antigenic classification of the CPV-2 and revealed the importance of the phylogenetic classification in the current dissemination of the virus (17, 20). Phylogenetically, our results corroborate the previous reports indicating that CPV-2 strains cluster into three major groups (GI, GII, GIII), demonstrating the heterogeneity and the distinct geographical distribution of CPV-2 and propose standard reference sequences to facilitate future genotyping classification and the prediction of new lineages. The comparative PI sites of NS1, NS2, and VP1/VP2 proteins make our proposed references more vigorous in terms of the distinct geographic characteristics of CPV-2.

Our genotyping classification system adds insights into Grecco et al. phylodynamic findings that were limited to 144 complete CPV-2 and CPV2a lineage genomes before 2018 without including African strains (17), and clarifies and updates the global distribution of all CPV-2 antigenic variants (a, b, and c) including strains collected before and after 2018. Grecco et al. showed that Eur-I includes European strains, South American (Argentina, Brazil, Ecuador, Paraguay, and Uruguay) strains, and strains from the United States. The Ecuadorian strains within the Eur-I clade clustered into a single branch and were associated with Southern European strains (17). The SA-I includes strains collected during 2003–2013 from Argentina and Brazil and a single strain collected in Uruguay during 2006. The Asia-I includes strains from China and India dated from 2004 to 2014 and Uruguayan strains collected during 2010–2011 (17). By using the proposed standard reference genomes, our report results revealed that Eur- I, Eur-II, and SA-I clades of the Grecco et al. study correspond to GI, the Asia-I clade correspond to GIII, while GII encompasses strains from Asia, particularly China, Italy, and Africa, which seems a new emerging lineage dominated by CPV-2c strains.

Furthermore, the South American strains (Argentina, Ecuador, and Brazil) classified into the GI seem to split within the clade into two subgroups following the reference standard: CPV-5.us.79 (GenBank ID: EU659116.1) detected in the USA in 1978 and are very probable to become two subclades. CPV-2 strains from Uruguay are identified in our phylogenetic trees related to Asia strains (China) in the GIII clade (GIII-b) and the USA and Europe strains in the GI clade, which might indicate different origins of Uruguay strains: from Asia and Europe or vice versa, which is concordant with Grecco et al. report (17).

Further, Grecco et al. have shown that the Asia I clade (Asia-I) encompasses Uruguay along with China and India strains and revealed characteristic residues at NS1 (272K) (17). The latter is detected as a PI site in our analysis. Even though Uruguay strains overlap with CPV-2 from multiple origins and demonstrate a close relationship with China strains and other Asian countries, Uruguay CPV-2 still has its unique molecular signature.

Besides, in China, the evolutionary relatedness between CPV-2s is still not fully clear. The analyses of fifty-two VP2 sequences, among which twenty-two strains were detected in domestic dogs between 1983 and 2008 compared to thirty other China sequences and eight non-China reference CPV-2 strains, have identified a high sequence identity among China variants (~98.3–100%) (28). Most of the strains detected in China clustered together (28). Consistently, our report indicates the distinct character and local adaptation of the strains in China that are restrictive to either GII or GIII clade. The NS1 and VP2 based-phylogenetic trees in Moon et al. report, comparing the first two identified CPV-2c in South Korea with sixty-one CPV-2 strains randomly selected from GenBank, showed that South Korea CPV-2c clustered separately from European, USA, and South American strains (29). Moon et al. demonstrated that South Korea CPV-2c was genetically closer to the Asian strains, sharing the maximum nucleotide similarity of 99.77%−99.34% with strains from China, Italy (detected in a dog imported from Thailand), and Vietnam (29). Therefore, a mutual transmission and circulation of CPV-2 between countries in Asia are highly suggested.

It should be noted that African CPV-2 strains, represented by Nigeria, cluster close to CPV-2 strains of Asian origins and belong to either GII or GIII (GIII-c). Further molecular epidemiological investigations are needed for better clarification.

The molecular epidemiology and genetic characteristics of CPV-2 in Italy know large gaps. The exploration of genetic features of CPV-2c strains currently spreading in Italy using the complete genome sequences analysis has shown a 99.95–99.91% nucleotide identities of CPV_IZSSI_2743_17 (GenBank ID: MF510157) from Italy with that of Canine/China/14/2017 (GenBank ID: MH476583) from China (30) and has identified identical amino acid changes with that of Asian CPV-2c strains in NS1(60V, 544F, 545F, 630P), NS2 (60V, 151N, 152V), and VP2(5A/G, 267Y, 297A, 324I, 370R) (30). However, CPV-2 strain IZSSI_PA5632/19 (GenBank ID: MK806285.1) revealed a supplemental amino acid substitution at NS1 protein (492 aa) (30), indicating the circulation of at least two different lineages of CPV-2 in Italy. In consistence, the standard reference sequences from Italy, in our phylogenetic tree, cluster into two distinct genotypes, including GI and GII. Therefore, our findings point out the divergence of Italy CPV-2 strains, which might be related to the intercontinental movement and migration of CPV-2. This pattern was also described by Mira et al. (30, 31).

Recombination is an important event in producing viral genetic diversity. The analysis of DNAs collected between 1978 and 2018 using deep sequencing has shown that over 40 years, all CPV-2 variants are homogeneous (~99%), and their evolution and spreading seem to depend mainly on early natural selection and the repair of DNA damage (27). Herein, the recombination analysis inferred that some of the circulating CPV-2a resulted from genetic recombination between only the newly identified CPV-2 strains, where CPV-2c serves as the major parent, indicating that genomic recombination might be behind the evolutionary processes and diversity of CPV-2. Therefore, a deep understanding and consideration of the detected recombination and the global movement may help achieve effective prevention and control of the new circulating CPV-2 variants.

Clinically, a correlation between CPV-2 viral topology and poor prognosis biological markers and disease severity was recently documented (32). CPV-2 virulence is suggested as an inherited feature where the pathogenicity can be transmitted from ancestors to descendant clusters (32). The prevention and control of CPV-2 spread and clinical infection depend mainly on the vaccination of the dog population. However, parvo virologists are still debating the vaccine efficacy since the initial CPV-2-based vaccines have been reported to be associated with decreased effectiveness against the current circulating antigenic CPV-2 strains (14). Increased CPV-2c outbreaks in vaccinated dogs have been reported recently (14) and might be related to discrepancies in amino acid substitutions and PI sites between strains from different geographical areas, the phylogenetic genotypes or the identified recombinants which may be involved in escaping the immune system and vaccine response.

The findings in this report would be efficient in inferring the relatedness of CPV-2 and suggest that CPV-2 appears to have multiple origins and are adapted and distinguished in a geographic manner. The sequence substitutions within NS1, NS2, and VP1/VP2 and recombinant strains identified here could mediate the specific evolution and adaptation of CPV-2 or affect the pathogenicity and transmissibility of the virus. For more effective research communication and better visualization and management of CPV-2 phylogenetic data, we initiate a set of standard reference sequences of the whole CPV-2 genome. Therefore, these findings would be useful to monitor the global dispersal of CPV-2, map its transmission pathways, and help achieve at least an improvement of the current CPV-2 situation.

## Data availability statement

The datasets presented in this study can be found in online repositories. The names of the repository/repositories and accession number(s) can be found in the article/Supplementary material.

## Author contributions

AB and LX conceived the study. AB and P-HW performed analysis. Z-HM and NR provided assistance in data analysis. AB wrote the manuscript. CW carried out administration. AB and LX revised the manuscript. All authors read and approved the final manuscript.

## Funding

The Programme of Introducing Talents of Discipline to Universities (D21004).

## Conflict of interest

The authors declare that the research was conducted in the absence of any commercial or financial relationships that could be construed as a potential conflict of interest.

## Publisher's note

All claims expressed in this article are solely those of the authors and do not necessarily represent those of their affiliated organizations, or those of the publisher, the editors and the reviewers. Any product that may be evaluated in this article, or claim that may be made by its manufacturer, is not guaranteed or endorsed by the publisher.
