# Supplementary material for: Identification of novel recombinants and proposed standard reference genomes for phylogenetic classification of canine parvovirus-2 (CPV-2): Comprehensive analysis revealing global evolutionary trait
Source: Front Vet Sci. 2022 Nov 15;9:1030522. doi: 10.3389/fvets.2022.1030522 (PMC9705586; doi:10.3389/fvets.2022.1030522)
Supplement: Supplementary file 1 [file Data_Sheet_1.pdf]

Supplementary Table

Identification of Novel Recombinants and Proposed Standard Reference Genomes for Phylogenetic Classification of Canine Parvovirus-2 (CPV-2): Comprehensive Analysis Revealing Global Evolutionary Trait

Amina Nawal Bahoussi [1], Pei-Hua Wang[1], Zi-Hui Ma[1], Nikita Rani[1], Changxin Wu[1,2,3,4], Li Xing[1,2,3,4]*

Supplementary Table 1. Genotype and subtype of Canine Parvovirus type 2 strains isolated from other 14 countries including Iran, Japan, India, Thailand, Mongolia, Bangladesh, Singapore, Australia, New Zealand, France, Albania, Germany, Finland, and Russia.

GenBank ID	Virus	Country	Continent	Year of collection	Antigenic Subtype	Genotype
MW653255.1	CPV/8/Iran	Iran	Middle East	2020	2a	GI
MW653254.1	CPV/18/Iran	Iran	Middle East	2020	2b	GI
MW653256.1	2a/6/Iran	Iran	Middle East	2020	2a	GI
MW653253.1	CPV/19/Iran	Iran	Middle East	2020	2c	GI
MW653252.1	CPV/30/Iran	Iran	Middle East	2020	2b	GI
MW653251.1	CPV/23/Iran	Iran	Middle East	2020	2b	GI
MW653250.1	CPV/22/Iran	Iran	Middle East	2020	2a	GIII-a
MW653249.1	CPV/2/Iran	Iran	Middle East	2020	2a	GIII-a
MW653248.1	CPV/Iran	Iran	Middle East	2020	2a	GIII-b
LC270891.1	9985	Japan	Asia	2017	2b	GI
LC270892.1	9985-46	Japan	Asia	2017	2b	GI
D26079.1	Y1	Japan	Asia	1993	2a	GI
AJ564427.2	CPV2a	India	Asia	1999	2a	GI
MH711902.1	CU21	Thailand	Asia	2016	2c	GII
MH711894.1	CU24	Thailand	Asia	2016	2c	GII
MH660909.1	5 MGL	Mongolia	Asia	2017	2c	GII
MT629886.1	CVASU parvovirus1	Bangladesh	Asia	2018	2a	GIII-a
MN661243.1	CPV new 2a variant	India	Asia	2016	2a	GIII-a
MH545963.1	TN/CPV2a/2018	India	Asia	2018	2a	GIII-a
KX618915.1	2a	Singapore	Asia	2016	2a	GIII-a
KF366250.1	CPV/915-H	India	Asia	2013	2a	GIII-b
KU508692.1	FH	Australia	Oceania	2015	2c	GI
KU508693.1	LW	Australia	Oceania	2015	2c	GI
KU508691.1	HB	Australia	Oceania	2015	2c	GI
MN451670.1	CPV87	Australia	Oceania	1985	2a	GI
MN451669.1	CPV81	Australia	Oceania	1982	2a	GI
AY742933.1	CPV-339	New Zealand	Oceania	1994	2a	GI
MF177227.1	202-09	France	Europe	2009	2c	GI
MN451666.1	CPV54	France	Europe	1984	2b	GI
MN451667.1	CPV58	France	Europe	1983	2a	GI
MF177229.1	368-12-17	Albania	Europe	2012	2c	GI
AY742934.1	CPV-447	Germany	Europe	1995	2b	GI
AY742935.1	CPV-U6	Germany	Europe	1995	2a	GI
MN451694.1	RDPV123	Finland	Europe	1980	2	GI
MN451695.1	RDPV124	Finland	Europe	1986	2	GI
MN451693.1	RDPV122	Finland	Europe	1980	2	GI
JN033694.1	Laika-1993	Russia	Europe	1993	2b	GI
