# Supplementary material for: Identification of novel recombinants and proposed standard reference genomes for phylogenetic classification of canine parvovirus-2 (CPV-2): Comprehensive analysis revealing global evolutionary trait
Source: Front Vet Sci. 2022 Nov 15;9:1030522. doi: 10.3389/fvets.2022.1030522 (PMC9705586; doi:10.3389/fvets.2022.1030522)
Supplement: Supplementary file 2 [file Presentation_1.pdf]

Supplementary Figure Legends

Identification of Novel Recombinants and Proposed Standard Reference Genomes for Phylogenetic Classification of Canine Parvovirus-2 (CPV-2): Comprehensive Analysis Revealing Global Evolutionary Trait

Amina Nawal Bahoussi [1], Pei-Hua Wang[1], Zi-Hui Ma[1], Nikita Rani[1], Changxin Wu[1,2,3,4], Li Xing[1,2,3,4]*

Supplementary Figures 1A-1D. Country-based phylogenetic trees of the entire genome sequences of CPV-2 isolates. Maximum-likelihood (ML)-based trees of CPV-2 isolates from four main countries: China (n= 62) **(A)**, Italy (n= 50) **(B)**, Uruguay (n= 48) **(C)**, and the USA (N= 47) **(D)**. Trees were generated using MEGA-11 software (Kumar et al., 2018; Tamura et al., 2004). Three main clades in phylogenetic trees of China, Italy and Uruguay are shown (clade1, 2 and 3), and two main clades (clades 1 and 2) in the USA. The standard references correspond to representative CPV-2 strains from each phylogenetic tree clade were indicated with Square (China), Diamond (Italy), Triangle (Uruguay), or Circle (USA). Blue, red, and green colors indicate strains belonging to GI, GII, and GIII, respectively. The numbers at each branch indicate the percentage of bootstrap values of 1000 replicates. The evolutionary distances were computed using the Maximum Composite Likelihood method. The scale bars reveal the number of inferred substitutions per site. The CPV-2 isolates are identified in a format as [GenBank ID_virus name (country-collection year-antigenic type in GenBank)].

References

Kumar, S., Stecher, G., Li, M., Knyaz, C., and Tamura, K. (2018). MEGA X: Molecular Evolutionary Genetics Analysis across Computing Platforms. Mol Biol Evol *35*, 1547-1549.

Tamura, K., Nei, M., and Kumar, S. (2004). Prospects for inferring very large phylogenies by using the neighbor-joining method. Proc Natl Acad Sci U S A *101*, 11030-11035.

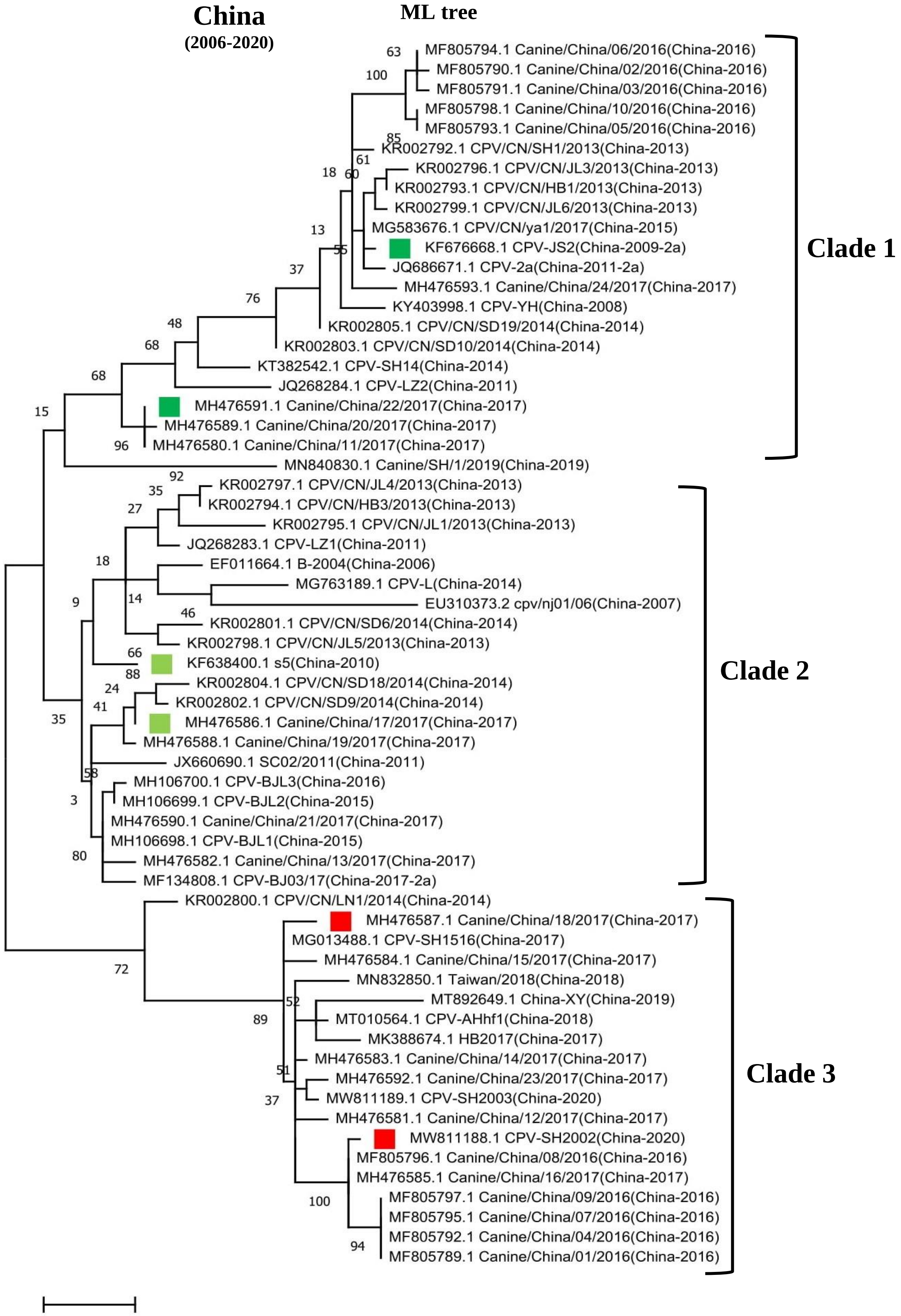

Supplementary Figure 1A

Italy
(1997-2019)

ML tree

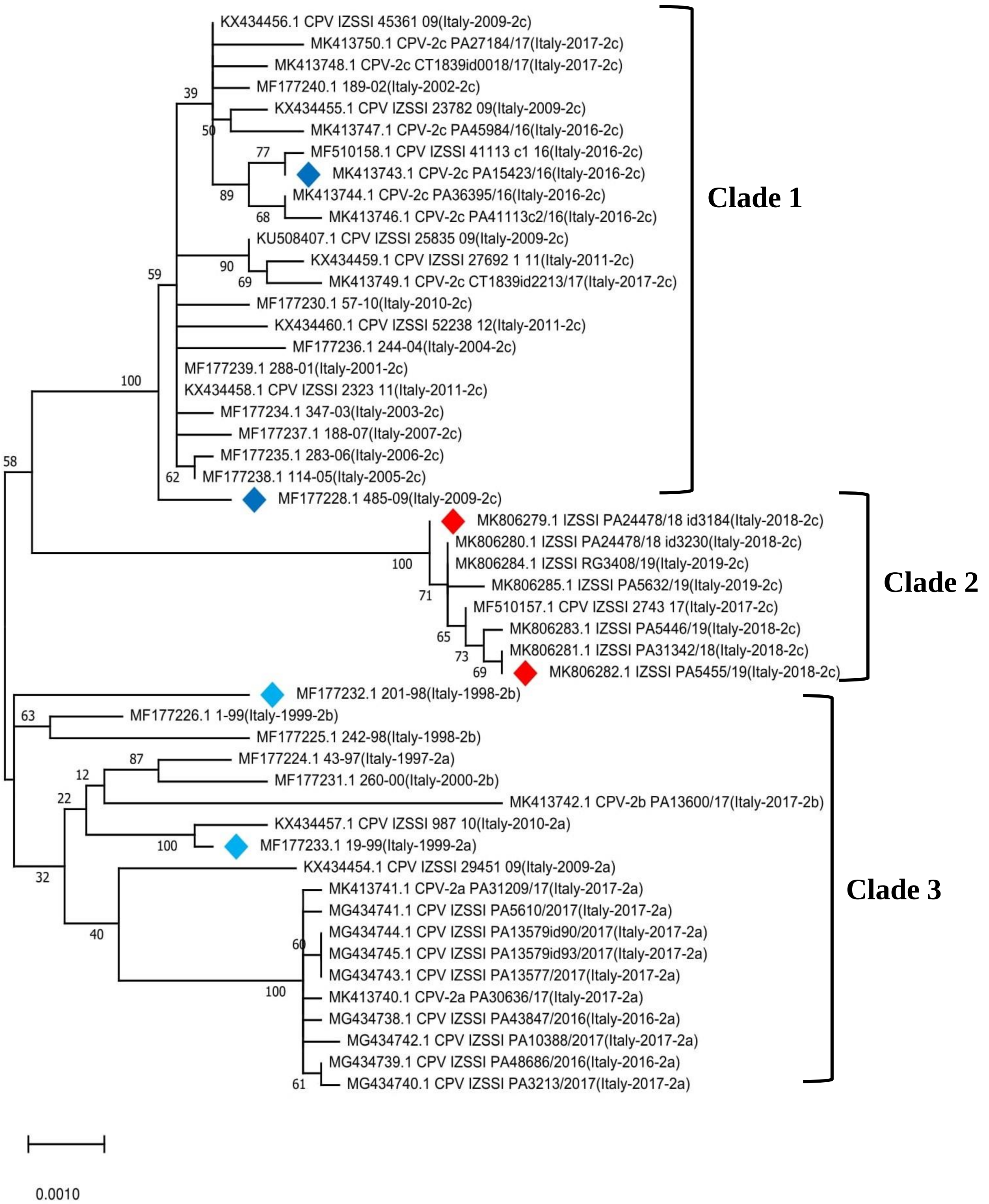

Supplementary Figure 1B

Uruguay
(2006-2013)

ML tree

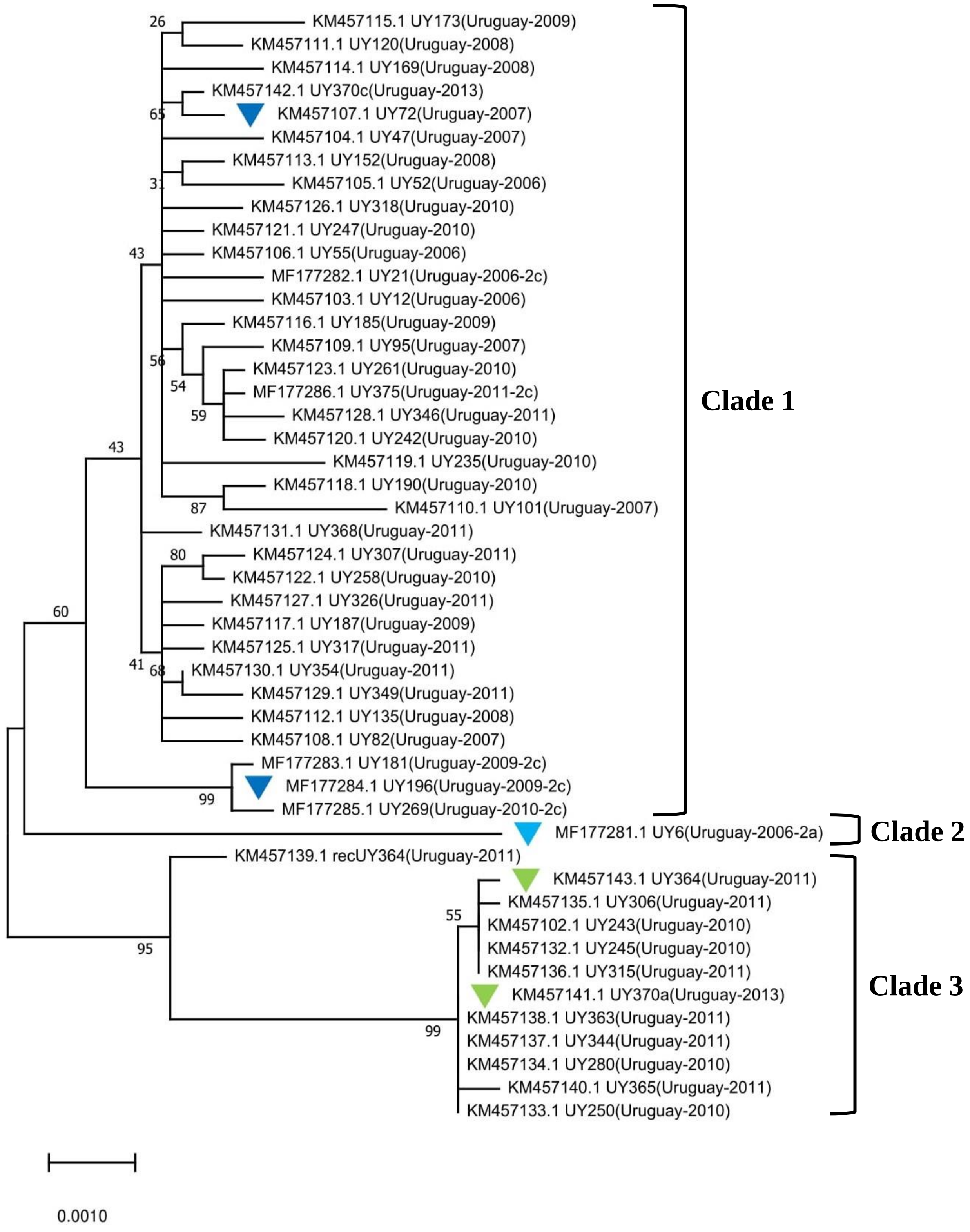

Supplementary Figure 1C

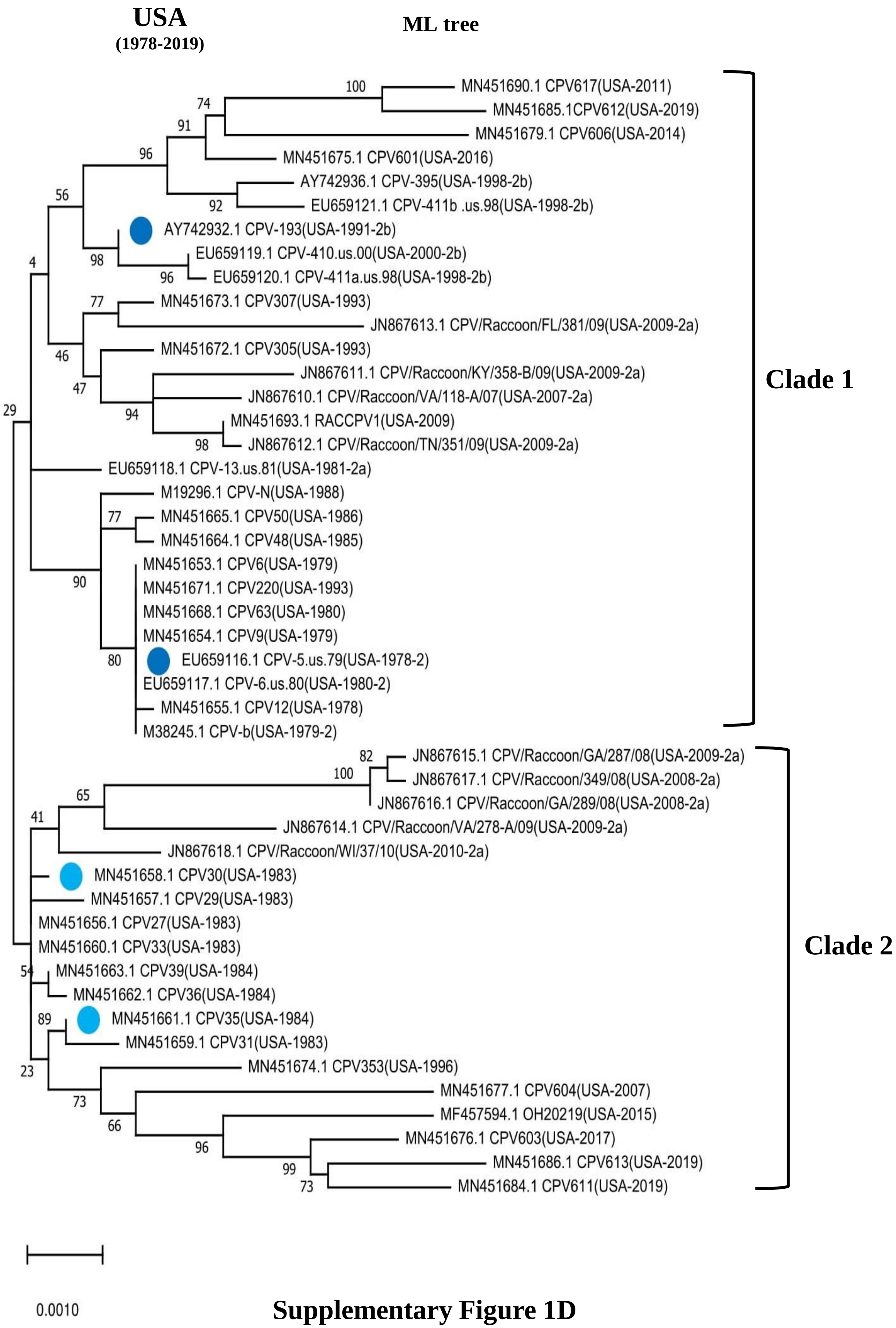

Supplementary Figure 1D
